# Population Pharmacokinetic and Pharmacodynamic Analysis To Evaluate a Switch to Doravirine/Lamivudine/Tenofovir Disoproxil Fumarate in People Living with HIV-1

**DOI:** 10.1128/AAC.00590-20

**Published:** 2020-10-20

**Authors:** Pavan Vaddady, Bhargava Kandala, Ka Lai Yee

**Affiliations:** a Merck & Co., Inc., Kenilworth, New Jersey, USA

**Keywords:** doravirine, fixed-dose combination, non-nucleoside reverse transcriptase inhibitor, population pharmacokinetic model, switch treatment

## Abstract

Doravirine is a non-nucleoside reverse transcriptase inhibitor for treatment of human immunodeficiency virus type 1 (HIV-1) infection. A population pharmacokinetic (PK) model for treatment-naive participants in doravirine clinical studies was updated with data from switch participants in the DRIVE-SHIFT trial and used to estimate individual *post hoc* PK parameter values and evaluate the efficacy exposure-response relationship. The results support the 100-mg dose for people living with HIV switching to a doravirine-based regimen (This study has been registered at ClinicalTrials.gov under ClinicalTrials registration no. NCT02397096.)

## Abstract (precis)

Doravirine is a non-nucleoside reverse transcriptase inhibitor for treatment of human immunodeficiency virus type 1 (HIV-1) infection. A population pharmacokinetic (PK) model for treatment-naive participants in doravirine clinical studies was updated with data from switch participants in the DRIVE-SHIFT trial and used to estimate individual *post hoc* PK parameter values and evaluate the efficacy exposure-response relationship. The results support the 100-mg dose for people living with HIV switching to a doravirine-based regimen (This study has been registered at ClinicalTrials.

## TEXT

People living with HIV require lifelong antiretroviral (ARV) treatment and may switch between several ARV regimens due to poor adherence or adverse events (1, 2). Doravirine (DOR) is a non-nucleoside reverse transcriptase inhibitor (NNRTI) available as single entity (3) and as a fixed-dose combination with nucleoside/nucleotide reverse transcriptase inhibitors (NRTIs) lamivudine (3TC) and tenofovir disoproxil fumarate (TDF). DOR/3TC/TDF is approved for the treatment of HIV-1 infection in adults who have not received prior ARV treatment or who are virologically suppressed on a stable ARV regimen that can be appropriately replaced by DOR/3TC/TDF (4). DOR is generally well tolerated in humans, with no relevant drug-related adverse events (5–8).

DRIVE-SHIFT (7) (protocol MK-1439A-024; ClinicalTrials registration no. NCT02397096), a phase 3, open-label, randomized, active-controlled, noninferiority trial in virologically suppressed participants with HIV-1, evaluated a switch from a stable regimen of two NRTIs plus a ritonavir- or cobicistat-boosted protease inhibitor, cobicistat-boosted elvitegravir, or NNRTI to DOR/3TC/TDF. Participants were randomized (2:1) to switch to DOR/3TC/TDF on day 1 (immediate switch group [ISG]) or to continue their baseline regimen until week 24 and then switch to DOR/3TC/TDF (delayed switch group [DSG]).

The primary endpoint in DRIVE-SHIFT was the proportion of participants with an HIV-1 RNA level of <50 copies/ml (U.S. Food and Drug Administration [FDA] snapshot approach), with the primary comparison between ISG at week 48 and DSG at week 24. The main objectives of the current analysis were to evaluate the consistency of DOR pharmacokinetics (PK) in the ISG population with that of the treatment-naive population described previously (9) and to evaluate the exposure-response relationship between different quantiles of DOR exposure and the primary endpoint in ISG participants to further inform on the efficacy and appropriateness of a switch to a DOR-based regimen.

A total of 670 participants on stable ARV regimens were recruited to the DRIVE-SHIFT trial (ISG, *N *= 447; DSG, *N *= 223) (7). DOR PK samples were collected from all participants on day 1 (predose) and week 48 (predose and within 0.5 to 2 h postdose). Additional ISG PK samples were collected at weeks 4 (predose), 12 (irrespective of dosing), and 24 (predose and 0.5 to 2 h postdose). Only PK data from the ISG were included in the population PK analysis, given sparse sampling in the DSG (week 48 only).

To evaluate consistency of DOR PK in the phase 3 switch population with that of the phase 3 treatment-naive population, several approaches (described in Text S1 in the supplemental material) were evaluated. The exploratory analyses (approach 1) and the estimation of the combined model (approach 3b) are presented here. Observed data from DRIVE-SHIFT were initially compared with observed data from treatment-naive participants within phase 3 trials (MK-1439-018 [P018], DRIVE-FORWARD, ClinicalTrials registration no. NCT02275780; MK-1439A-021 [P021], DRIVE-AHEAD, ClinicalTrials registration no. NCT02403674). A previously described population PK model for the treatment-naive population (9) was updated in the current analysis using DOR concentration data from the ISG of DRIVE-SHIFT. Population PK parameters, including covariates, were reestimated, and the final model was used to estimate individual *post hoc* PK parameter values for the switch and treatment-naive populations.

The population PK analysis data set comprised the original 341 healthy participants and 959 treatment-naive participants with HIV-1, with the addition of 443 virologically suppressed participants with HIV-1 from the DRIVE-SHIFT ISG (a total of 1,402 participants with HIV-1) (9). Four of 447 ISG individuals were excluded due to data reconciliation issues. Comparison of PK data from treatment-naive participants in prior phase 3 studies with those from DRIVE-SHIFT suggested a comparable range of DOR concentrations at different steady-state time points (see Fig. S1 in the supplemental material) and indicated the suitability of the previously developed population PK model for the DRIVE-SHIFT ISG population PK analysis (7).

The original DOR population PK model was a one-compartment model with first-order absorption and linear apparent clearance (CL/F). Body weight and healthy versus HIV-1 infection status were covariates on apparent volume of distribution and age on apparent clearance. This model characterized the ISG data well, as supported by the diagnostic plots (see Text S1, Fig. S2, and Table S1 in the supplemental material).

The final PK parameters of the model (Table S1) were well estimated, with small standard errors. These estimates were very similar to those of the previously developed population PK model based only on data from healthy subjects and treatment-naive participants with HIV-1 (9).

**FIG 1:**
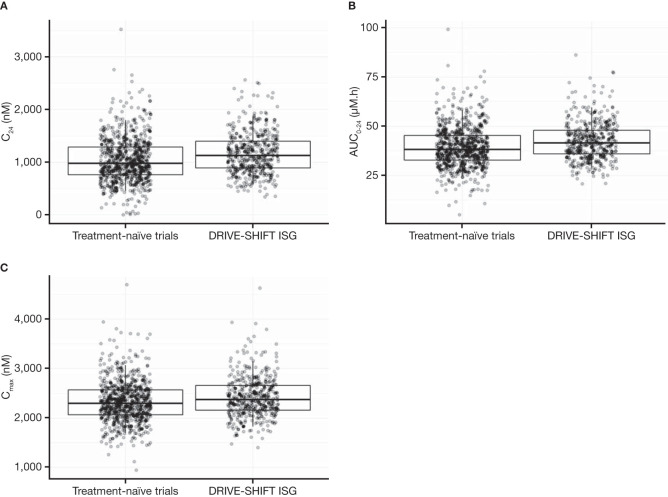
Comparison of steady-state DOR (A) *C*_24_, (B) AUC_0–24_, and (C) *C*_max_ following administration of 100 mg once-daily DOR between treatment-naive trials (P018 and P021) and participants from the ISG of DRIVE-SHIFT (P024). Boxes represent 25th, 50th, and 75th percentiles. Whiskers represent the 5th and 95th percentiles of the respective distributions for *C*_24_, AUC_0–24_, or *C*_max_. AUC_0–24_, area under the concentration-time curve from 0 to 24 h; *C*_24_, plasma concentration 24 h after dose administration; *C*_max_, maximum serum drug concentration; DOR, doravirine; ISG, immediate switch group.

PK parameters, including area under the concentration-time curve from 0 to 24 h (AUC_0–24_), maximum serum concentration (*C*_max_), and plasma drug concentration 24 h after dose administration (*C*_24_) at steady state, were simulated from *post hoc* compartmental parameter estimates for each participant in the phase 3 studies. Table 1 and Fig. 1 show the distributions of individual steady-state AUC_0–24_, *C*_max_, and *C*_24_ from the DRIVE-SHIFT ISG are comparable to those in treatment-naive phase 3 studies (P018 and P021).

**TABLE 1:** Summary statistics of DOR steady-state AUC_0–24_, *C*_max_, and *C*_24_ following administration of once-daily 100 mg DOR in treatment-naive study participants and participants randomized to the ISG in the DRIVE-SHIFT trial (P024)^*a*^

Phase 3 study population	Dose (mg)	Parameter	*N*	Geometric mean	Geometric % CV
Treatment naive (P018 and P021)	100	AUC_0–24_ (μM · h)	730	38.1	28.8
		*C*_24_ (nM)	730	932	62.7
		*C*_max_ (nM)	730	2,290	18.2
DRIVE-SHIFT ISG (P024)	100	AUC_0–24_ (μM · h)	443	41.5	22.8
		*C*_24_ (nM)	443	1,110	36.1
		*C*_max_ (nM)	443	2,390	16.5

a AUC_0–24_, area under the concentration-time curve from 0 to 24 h; *C*_24_, plasma concentration 24 h after dose administration; *C*_max_, maximum serum drug concentration; CV, coefficient of variation; DOR, doravirine; ISG, immediate switch group; *N*, number of participants.

The AUC_0–24_, *C*_max_, and *C*_24_ estimates for the DRIVE-SHIFT ISG were used in efficacy exposure-response exploratory analyses for this population. The efficacy endpoints used were the proportion of ISG participants maintaining HIV-1 RNA levels of <50 copies/ml and <40 copies/ml at week 48 (yes/no). Analyses were conducted for (i) the primary snapshot approach specified in the phase 3 trial protocols that classified any participant with missing data as a failure and (ii) the observed failure approach, where monotone (nonintermittent) missing data for participants who discontinued treatment prematurely due to lack of efficacy were assigned as failures after discontinuation, whereas those with missing data for other reasons were excluded.

The exposure-response analysis data set included virological response data from 443 individuals. A linear exposure-effect model performed very similarly to the log exposure-effect model based on the Akaike Information Criterion values; hence the linear model was chosen for the analyses. The slope and 95% confidence interval (CI) of the exposure-response relationship were estimated. A *P* value was calculated for the slope to evaluate whether it was significant (nonzero) or insignificant (not different from zero).

**FIG 2:**
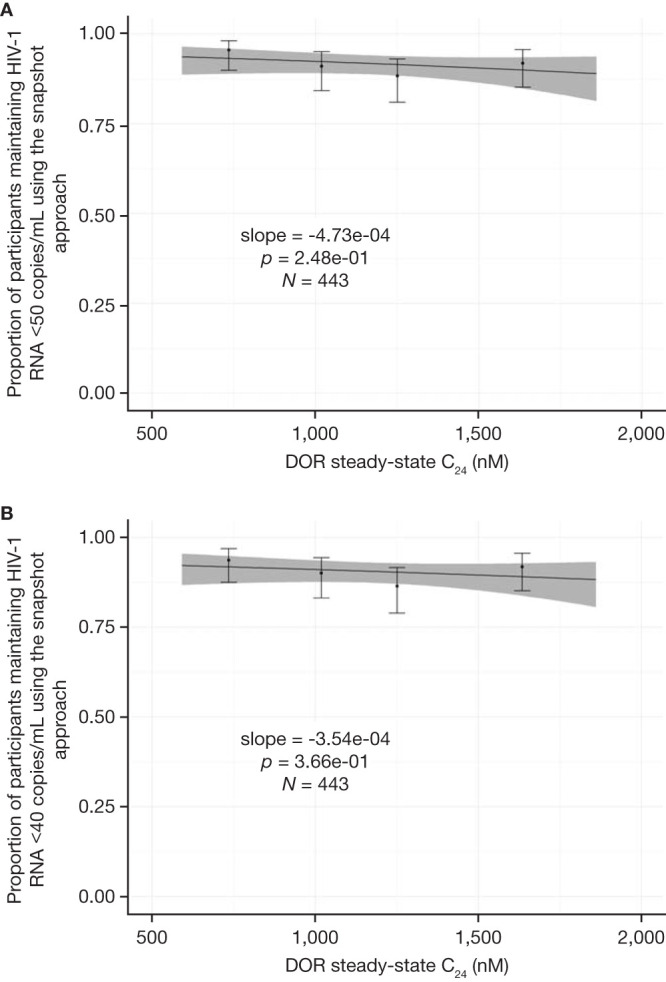
Predicted and observed proportion of ISG participants maintaining HIV-1 RNA at (A) <50 copies/ml or (B) <40 copies/ml using the snapshot approach as a function of DOR steady-state *C*_24_ quartiles following administration of 100 mg once-daily DOR (*N *= 443). Solid lines signify the mean predicted exposure-response relationship. Shaded areas represent the 95% CI of the prediction over the 5th to 95th percentiles of exposures. Markers and whiskers summarize the observed endpoint and 95% CI by *C*_24_ quantile. AUC_0–24_, area under the concentration-time curve from 0 to 24 h; *C*_24_, plasma concentration 24 h after dose administration; *C*_max_, maximum serum drug concentration; DOR, doravirine; ISG, immediate switch group.

Slope estimates from the exposure-response analyses were not significantly different from zero (*P* values of >0.05), suggesting a flat exposure-response relationship with no trends between virologic response and DOR exposure over the range of exposures achieved with once-daily 100-mg doses in the DRIVE-SHIFT ISG. Consequently, structural models of increased complexity were not explored and no covariate analysis was performed. Figure 2 shows the exposure-response relationships with DOR steady-state *C*_24_ (snapshot approach).

As DOR steady-state PK and exposure-response relationship are the same between treatment-naive and switch populations, the DOR clinical pharmacology profile characterized in the treatment-naive population, including the effects of intrinsic factors and drug-drug interactions, is also applicable to the switch population. In patients switching from efavirenz, a moderate cytochrome P450 (CYP) 3A inducer, plasma concentrations of DOR may be transiently decreased as the induction effects of efavirenz are washed out (10, 11). However, the efficacy and PK profile of DOR in participants switching from efavirenz were found to be similar to those of participants switching from other ARV therapies (10). From a physiological perspective, sustained virologic suppression is not anticipated to impact the PK of DOR, consistent with the findings of this analysis. The similarity of DOR PK between treatment-naive and switch populations indicates that dose recommendations for DOR determined in the treatment-naive population are directly applicable to the switch population without adjustments and supports the appropriateness of the 100-mg dose of DOR within the switch population.

## Data availability.

The data sharing policy of Merck Sharp & Dohme Corp., a subsidiary of Merck & Co., Inc., Kenilworth, NJ, including restrictions, is available at http://engagezone.msd.com/ds_documentation.php. Requests for access to the clinical study data can be submitted through the EngageZone site or via email to dataaccess@merck.com.

## Supplementary Material

Supplemental file 1

## References

[1] Thompson MA, Mugavero MJ, Amico KR, Cargill VA, Chang LW, Gross R, Orrell C, Altice FL, Bangsberg DR, Bartlett JG, Beckwith CG, Dowshen N, Gordon CM, Horn T, Kumar P, Scott JD, Stirratt MJ, Remien RH, Simoni JM, Nachega JB 2012 Guidelines for improving entry into and retention in care and antiretroviral adherence for persons with HIV: evidence-based recommendations from an International Association of Physicians in AIDS Care panel. Ann Intern Med 156:817–833. doi:10.7326/0003-4819-156-11-201206050-00419. 22393036 PMC4044043

[2] Nachega JB, Mugavero MJ, Zeier M, Vitória M, Gallant JE 2011 Treatment simplification in HIV-infected adults as a strategy to prevent toxicity, improve adherence, quality of life and decrease healthcare costs. Patient Prefer Adherence 5:357–367. doi:10.2147/PPA.S22771. 21845035 PMC3150164

[3] Merck Sharp & Dohme Corp. 2019 PIFELTRO (doravirine) prescribing information. Merck & Co, Inc, Whitehouse Station, NJ https://www.merck.com/product/usa/pi_circulars/p/pifeltro/pifeltro_pi.pdf. Accessed 16 March 2020.

[4] Merck Sharp & Dohme Corp. 2019 DELSTRIGO (doravirine/lamivudine/tenofovir disoproxil fumarate) prescribing information. Merck Sharp & Dohme Corp, Whitehouse Station, NJ https://www.merck.com/product/usa/pi_circulars/d/delstrigo/delstrigo_pi.pdf. Accessed 16 March 2020.

[5] Gatell JM, Morales-Ramirez JO, Hagins DP, Thompson M, Arasteh K, Hoffmann C, Raffi F, Osiyemi O, Dretler R, Harvey C, Xu X, Plettenberg A, Smith DE, Portilla J, Rugina S, Kumar S, Frobose C, Wan H, Rodgers A, Hwang C, Teppler H 2019 Doravirine dose selection and 96-week safety and efficacy versus efavirenz in antiretroviral therapy-naive adults with HIV-1 infection in a Phase IIb trial. Antivir Ther 24:425–435. doi:10.3851/IMP3323. 31355775

[6] Orkin C, Squires KE, Molina JM, Sax PE, Wong WW, Sussmann O, Kaplan R, Lupinacci L, Rodgers A, Xu X, Lin G, Kumar S, Sklar P, Nguyen BY, Hanna GJ, Hwang C, Martin EA, DRIVE-AHEAD Study Group. 2019 Doravirine/lamivudine/tenofovir disoproxil fumarate is non-inferior to efavirenz/emtricitabine/tenofovir disoproxil fumarate in treatment-naive adults with human immunodeficiency virus-1 infection: week 48 results of the DRIVE-AHEAD trial. Clin Infect Dis 68:535–544. doi:10.1093/cid/ciy540. 30184165 PMC6355823

[7] Johnson M, Kumar P, Molina J-M, Rizzardini G, Cahn P, Bickel M, Mallolas J, Zhou Y, Morais C, Kumar S, Sklar P, Hanna GJ, Hwang C, Greaves W, DRIVE-SHIFT Study Group. 2019 Switching to doravirine/lamivudine/tenofovir disoproxil fumarate (DOR/3TC/TDF) maintains HIV-1 virologic suppression through 48 weeks: results of the DRIVE-SHIFT trial. J Acquir Immune Defic Syndr 81:463–472. doi:10.1097/QAI.0000000000002056. 30985556 PMC6905402

[8] Molina J-M, Squires K, Sax PE, Cahn P, Lombaard J, DeJesus E, Lai M-T, Xu X, Rodgers A, Lupinacci L, Kumar S, Sklar P, Nguyen B-Y, Hanna GJ, Hwang C, for the DRIVE-FORWARD Study Group 2018 Doravirine versus ritonavir-boosted darunavir in antiretroviral-naive adults with HIV-1 (DRIVE-FORWARD): 48-week results of a randomised, double-blind, phase 3, non-inferiority trial. Lancet HIV 5:e211–e220. doi:10.1016/S2352-3018(18)30021-3. 29592840

[9] Yee KL, Ouerdani A, Claussen A, de Greef R, Wenning L 2019 Population pharmacokinetics of doravirine and exposure-response in individuals with HIV-1. Antimicrob Agents Chemother 63:e02502-18. doi:10.1128/AAC.02502-18. 30745394 PMC6437494

[10] Greaves W, Wan H, Yee KL, Kandala B, Vaddady P, Hwang C 2019 Doravirine exposure and HIV-1 suppression after switching from an efavirenz-based regimen to doravirine/lamivudine/tenofovir disoproxil fumarate. Antimicrob Agents Chemother 63:e1298-19. doi:10.1128/AAC.01298-19. PMC6879224 31548188

[11] Yee KL, Sanchez RI, Auger P, Liu R, Fan L, Triantafyllou I, Lai MT, Di Spirito M, Iwamoto M, Khalilieh SG 2017 Evaluation of doravirine pharmacokinetics when switching from efavirenz to doravirine in healthy subjects. Antimicrob Agents Chemother 61:e01757-16. doi:10.1128/AAC.01757-16. PMC5278744 27872069

