# Supplementary material for: Population Pharmacokinetic and Pharmacodynamic Analysis To Evaluate a Switch to Doravirine/Lamivudine/Tenofovir Disoproxil Fumarate in People Living with HIV-1
Source: Antimicrob Agents Chemother. 2020 Oct 20;64(11):e00590-20. doi: 10.1128/AAC.00590-20 (PMC7577165; doi:10.1128/AAC.00590-20)
Supplement: Supplemental file 1 [file AAC.00590-20-s0001.pdf]

SUPPLEMENTAL MATERIAL

Population Pharmacokinetic and Pharmacodynamic Analysis to Evaluate a Switch to Doravirine/Lamivudine/Tenofovir Disoproxil Fumarate in People Living with HIV-1

Pavan Vaddady, Bhargava Kandala, Ka Lai Yee

Merck & Co., Inc., Kenilworth, NJ, USA

SUPPLEMENTAL METHODS

Model development

Given the sparse dataset from P024 ISG, several approaches were evaluated and only the final version is presented in the main publication. The following approaches were evaluated:

1. Exploratory plots comparing observed data from treatment naïve with ISG and DSG groups. These results are presented in the main and supplemental sections of the publication.

2. Observed PK data from P024 overlaid on the simulations based on P024 demographic data and treatment information utilizing the prior DOR population PK model. These results indicated that the prior model is able to explain the new data and these results are not shown in the publication.

3. With the population PK dataset containing all the PK data including data from immediate switch arm in P024:

a. A single estimation run in NONMEM with MAXEVAL=0 was carried out using the prior final PK model with the exact model structure, including covariates and error structures. These results are not shown in the publication.

b. An estimation run in NONMEM was carried out using the prior final DOR population PK model structure including the covariates as a starting model. With this final model, resulting PK exposure values from treatment-naïve participants were compared to the treatment-experienced participants using graphical displays and tabulated statistical summaries. These results are presented in the main and supplemental sections of the publication.

Empirical Bayes estimates (EBEs) from a and b were compared to evaluate the consistency of PK in the treatment-experienced populations with that of the treatment-naïve population. The results indicated similar EBEs between these groups.

4. A covariate for treatment-naïve versus treatment-experienced was applied to key PK parameters (CL and V) and statistical significance for this covariate was evaluated to check for the consistency in PK between treatment-naïve and treatment-experienced populations. This was found to be insignificant.

Logistic Regression Models

Logistic regression models were used to quantify the relationship between the likelihood of maintaining the endpoint and the steady-state PK parameters. A base structural model was assessed to find a best fit for the exposure–response relationship. The linear model was initially explored (i), and subsequently models of increasing complexity were considered (ii) in case of significant exposure–response trends.

i) Logistic regression with linear exposure (e.g., with C_{24} as exposure measure)

$$logit(p) = \log\left(\frac{p}{1-p}\right) = b_0 + b_1\,C24 \;\Leftrightarrow p = \frac{e^{b_0+b_1C24}}{1+e^{b_0+b_1C24}}$$

ii) Logistic regression with log-transformed exposure (e.g., with C_{24} as exposure measure)

$$logit(p) = \log\left(\frac{p}{1-p}\right) = b_0 + b_1 \log{(C24)} \Leftrightarrow p = \frac{e^{b_0 + b_1 \log{(C24)}}}{1 + e^{b_0 + b_1 \log{(C24)}}}$$

The goodness of fit of the final models was assessed by overlaying the observed responses with the model-predicted responses across quantiles of exposure and by numerical predictive check, where simulated proportions of events and observed proportions were compared across quantiles of exposure.

SUPPLEMENTAL RESULTS

Supplemental Figure 1

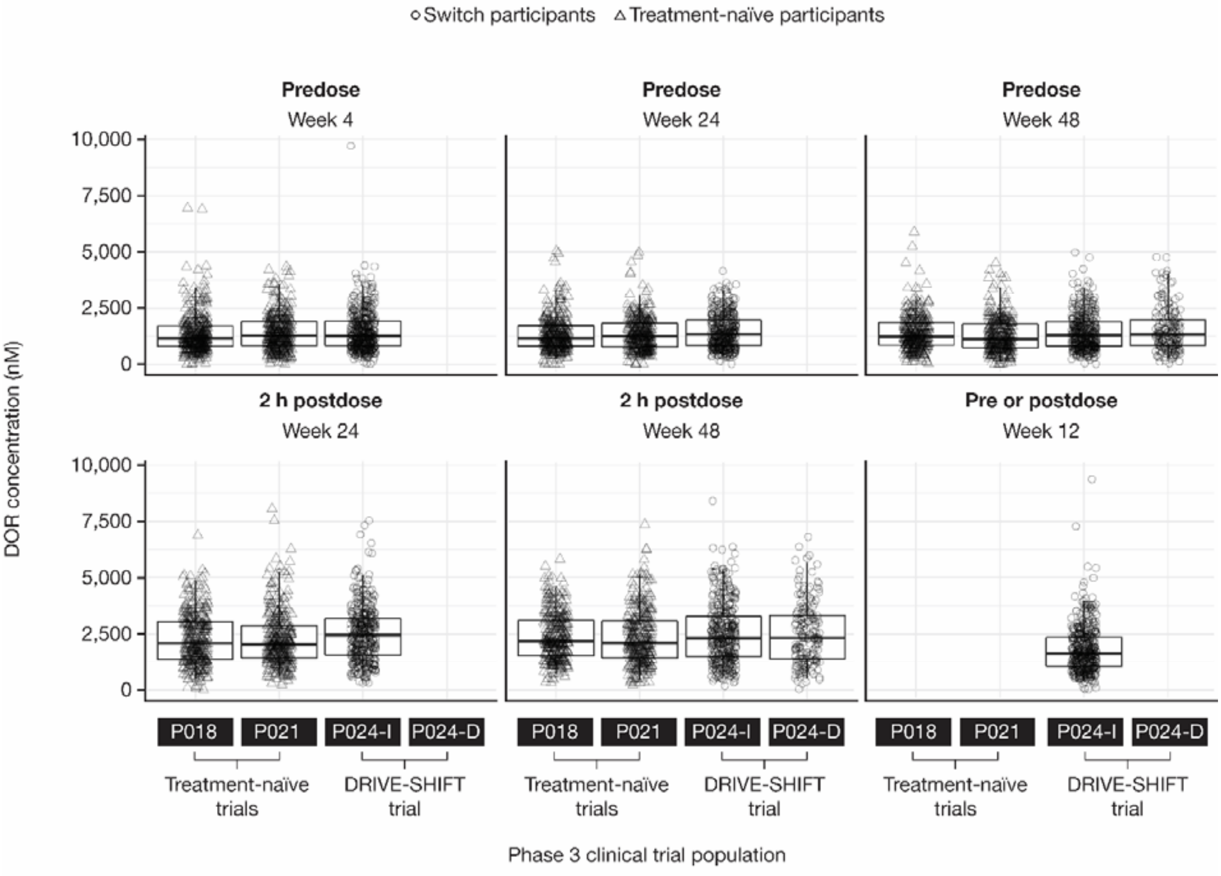

Supplemental Figure 1

Exploratory PK analysis comparing Phase 3 DOR PK data from treatment-naïve participants (P018 and P021) and DRIVE-SHIFT participants (P024).

Boxplots with 95% confidence intervals overlaid with individual observed data points.

D, delayed switch group; DOR, doravirine; h, hours; I, immediate switch group.

Scatter Plots of ISG Data

Scatter plots of population-predicted and individual-predicted versus observed concentrations showed an even distribution around the line of unity (Supplemental Figure 2). Moreover, scatter plots of conditional weighted residuals (CWRES) versus population-predicted concentrations and of CWRES versus time showed the CWRES to be evenly distributed around zero, indicating the validity of the use of the previously described population PK model.

Supplemental Figure 2

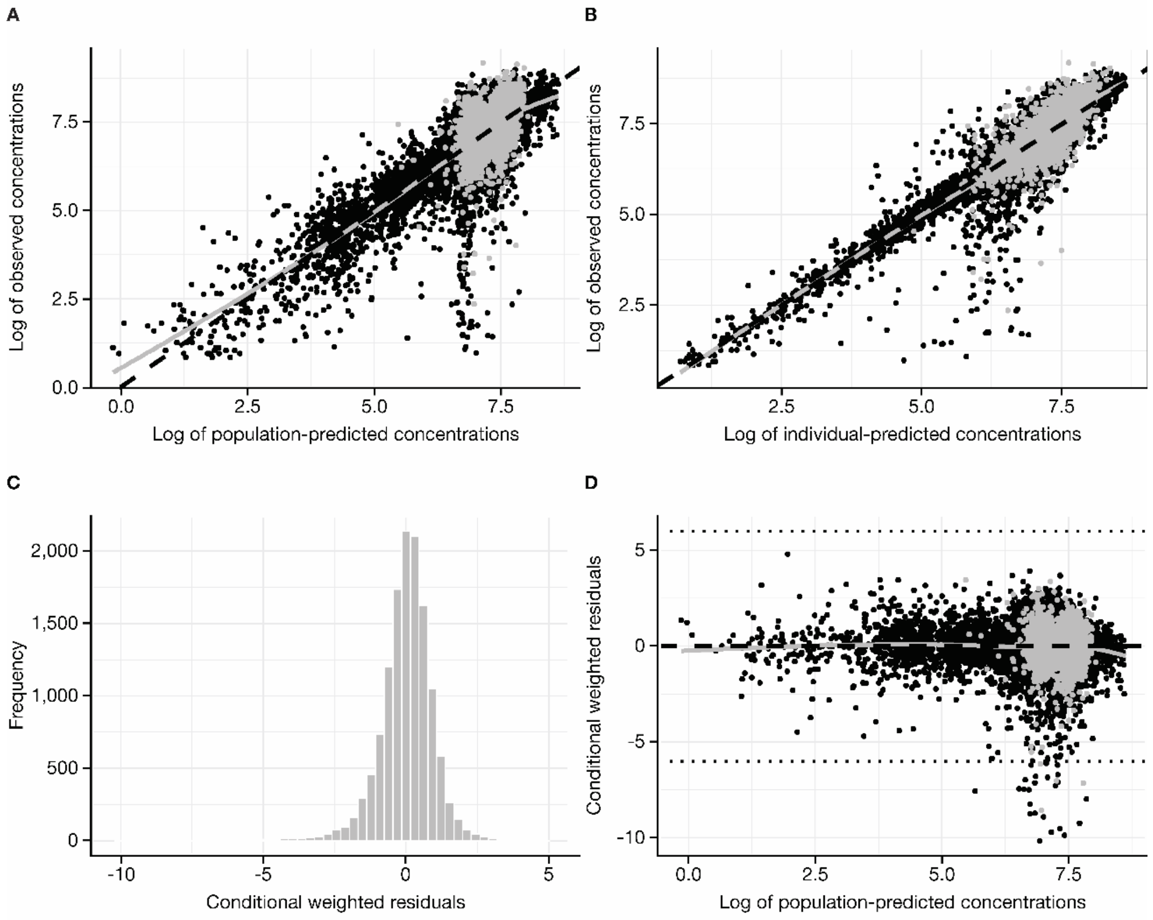

Supplemental Figure 2
Goodness-of-fit plots of the final model.
Solid gray lines in (A), (B), and (D) represent the Locally Weighted Scatterplot Smoothing smoothed line. In (A) and (B), black dashed lines signify the line of unity.
In (D), a black dashed line signifies conditional weighted residuals = 0. Black dotted lines in (D) represent limits defining outlier observations (-6, 6).
Black dots in (A), (B), and (D) represent individual participants from treatment-naïve trials.
Gray dots represent individuals from the DRIVE-SHIFT immediate switch group.

SUPPLEMENTAL TABLE 1 Parameter estimates for the final population pharmacokinetic model

	**Fixed effects**					
	**Final estimate**	**%RSE**	**%CV**	**95% LBOUND**	**95% UBOUND**	
**CL/F (L/h)**	6.21	1.19	—	6.07	6.40	
**V/F (L)**	159	2.72	—	151	168	
**K_a (1/h)**	1.42	4.45	—	1.30	1.54	
**F1 <30 mg**	1.20	5.72	—	1.07	1.33	
**F1 30–120 mg (reference)**	1	—	—	—	—	
**F1 >120 mg**	0.882	9.10	—	0.725	1.04	
**Age on CL**	-0.00540	13.0	—	-0.00678	-0.00402	
**Subject status on V**	-0.205	12.1	—	-0.254	-0.156	
**Weight on V**	0.00788	12.9	—	0.00590	0.00986	
	**Interindividual variability**					
	**Final estimate**	**%RSE**	**%CV**	**95% LBOUND**	**95% UBOUND**	**% η-SHRINKAGE**
**ω^2CL/F**	0.104	6.95	33.1	0.0898	0.118	12.5
**ω^2V/F**	0.098	7.98	32.1	0.0828	0.114	43.1
	**Residual variability**					
	**Final estimate**	**%RSE**	**%CV**	**95% LBOUND**	**95% UBOUND**	**% ε-SHRINKAGE**
**SD Phase 1 ≤0.5 h postdose**	0.224	3.78	22.4	0.207	0.241	7.55
**SD Phase 1 >0.5 h postdose**	1.25	6.92	125	1.08	1.42	7.55
**SD Phase 2b/3**	0.504	3.18	50.4	0.473	0.535	7.55

95% confidence interval is: $\omega^2 \pm 1.96 \times SE\omega^2$

CV% for IIV: $\sqrt{(e^{\omega^2} - 1)}$ with final estimate equal to ω^2
CV% for residual variability: $\left(\frac{SD}{Mean}\right) \times 100$ with mean equal to 1
$CL_i = TVCL\left(1 + \theta\,(Age_i - 34)\right)$
$V_i = TVV\left(1 + \theta\,(Weight_i - 75)\right)$

ω^2, variance of the parameter distribution (final estimate of variability parameters); CL/F, apparent linear clearance; CV, coefficient of variation; F1, relative bioavailability; IIV, Inter-individual variability; K_a, absorption rate; LBOUND, lower two-sided confidence bound; RSE, relative standard error; SD, standard deviation; TVCL, typical value for apparent clearance; TVV, typical value of volume of distribution; V/F, apparent volume of distribution; UBOUND, upper two-sided confidence bound.
